# Gaucher Disease: An Unusual Cause of Knee Pain

**DOI:** 10.5435/JAAOSGlobal-D-21-00243

**Published:** 2022-10-11

**Authors:** Ioannis Gigis, Charalampos Pitsilos, Efthimios Samoladas, Charalampos Pavlopoulos, Prodromos Hytiroglou, Konstantinos Ditsios, Pericles Papadopoulos

**Affiliations:** From the 2nd Orthopaedic Department, Aristotle University of Thessaloniki, “G. Gennimatas” General Hospital, Thessaloniki, Greece (Gigis, Pitsilos, Pavlopoulos, Ditsios, and Papadopoulos), 1st Orthopaedic Department, Aristotle University of Thessaloniki, “G.Papanikolaou” General Hospital, Thessaloniki, Greece (Samoladas), and the Department of Pathology, School of Medicine, Aristotle University of Thessaloniki, Greece (Hytiroglou).

## Abstract

Gaucher disease (GD) is a genetic lysosomal disorder leading to storage of the glycolipid molecule glucocerebroside in macrophages, causing multiorgan dysfunction. Bone marrow involvement may result in painful bone crisis and hematologic disturbance.

**Case Report:**

We present a case of a 13-year-old adolescent boy with right knee pain. Radiograph and magnetic resonance imaging of the distal femur indicated possible osteomyelitis or bone tumor. However, histologic examination of bone biopsy material suggested the diagnosis of GD, which was confirmed by detection of decreased β-glucocerebrosidase activity and identification of the exact gene mutation.

**Discussion:**

Many visceral and bone abnormalities of GD have been described. The diagnosis of GD is based on clinical and laboratory findings and is established by the measurement of β-glucocerebrosidase dysfunction and the study of GBA gene mutations. Treatment is currently based on enzyme replacement and substrate reduction.

**Conclusion:**

This is a rare case of GD presenting initially with knee pain. Because early diagnosis is important for the treatment of this condition, orthopaedic surgeons should consider this uncommon cause in the differential diagnosis of joint pain.

Gaucher disease (GD) is the most common hereditary lysosomal storage disorder. It is an autosomal recessive genetic disease caused by a mutation in the GBA gene in chromosome 1, with more than 300 described variants, which was first described by Philippe Gaucher in 1882.^1^ The incidence of GD is around 1 in 50,000 to 100,000 births in general population, with a more frequent occurrence in the Ashkenazi Jewish population, reaching 1 in 850 births.^2^ GD is characterized by the deficiency of the enzyme β-glucocerebrosidase, also known as β-glucosidase. This results in the storage of its substrate, glucocerebroside, in macrophages, which accumulate mainly in the liver, spleen, bones, and bone marrow, causing multisystemic manifestations.^3^ These modified macrophages, called “Gaucher cells,” are typical in GD and can be detected in bone marrow aspirate and liver or spleen biopsy.^4^ Three types of GD have been described. Type 1 is characterized by hepatosplenomegaly and hematologic bone and bone marrow abnormalities in different combinations. Occasionally, peripheral neuropathy, pulmonary disease, malignancy predisposition, and immunologic disorders may develop. Type 2 presents in infants causing severe visceral and neurologic dysfunction and progresses to death by the age of 2 or 3 years. Type 3 appears in childhood, is mainly neurologically compromising, and responds to enzyme treatment with life expectancy reaching the fifth or sixth decade.^5^

In this article, we report a case of an adolescent with a painful knee joint as the initial symptom. The imaging and laboratory findings resulted in the diagnosis of GD. Knee pain is usual in young patients visiting orthopaedic surgeons. Acute onset and no history of trauma should increase suspicion for additional investigation. We presented this case because early diagnosis is important because GD is rare and adequate therapy is essential.

## Case Report

**Figure 1:**
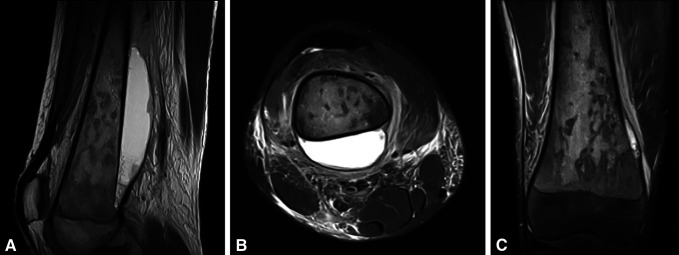
MRI of the right knee. Distal femur sagittal (**A**) and transverse (**B**) views, which demonstrate the large fluid collection on the posterior surface of the distal femur and diffuse soft-tissue edema. Distal femur coronal (**C**) view, which shows bone marrow heterogeneity and elevated metaphyseal periosteum.

A 13 year-old adolescent boy visited his family physician complaining of mild pain and swelling around his right knee. In the suspicion of a cutaneous infection, he was prescribed oral cefuroxime and analgesics. Because the pain and edema were gradually worsening, MRI of his right knee was conducted illustrating a fluid collection on the posterior aspect of the distal femur, diffuse soft-tissue edema, elevated metaphyseal periosteum, and mild bone marrow heterogeneity (Figure 1, A–C). These findings were compatible with the diagnosis of osteomyelitis or bone tumor.

**Figure 2:**
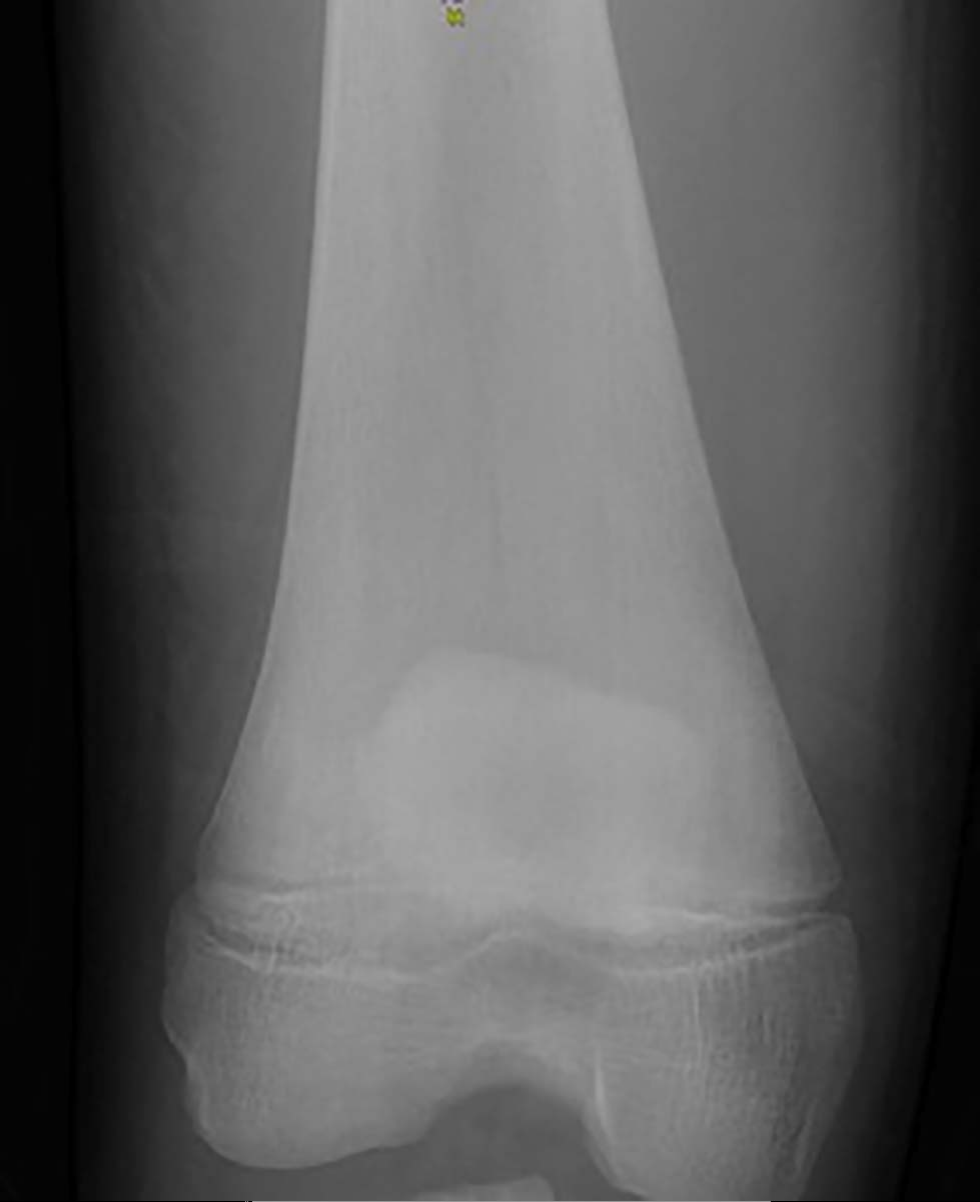
Radiograph showing the Erlenmeyer flask deformity of the distal femur.

Therefore, the patient was referred to our orthopaedic department for additional investigation and adequate treatment. He denied any recent injury, and he was free of any known hereditary or acquired disease. His parents also had no remarkable medical history. Physical examination revealed a significantly swollen right knee with painful, restricted range of motion. The body temperature was normal; however, the patient reported low-grade fever (37.4°C) on the day before admission. His blood test results were notable for anemia (hemoglobulin: 10.9 gr/dL, hematocrit: 32%) and an elevated erythrocyte sedimentation rate (55 mm/hr, normal: <10 mm/hr) and C-reactive protein (1.65 mg/dL, normal: <0.5 mg/dL). Distal right femur and knee radiographs were notable of the “Erlenmeyer flask deformity” of the distal femur (Figure 2). His right lower extremity was immobilized in a posterior long leg splint, and he was hospitalized for further investigation.

**Figure 3:**
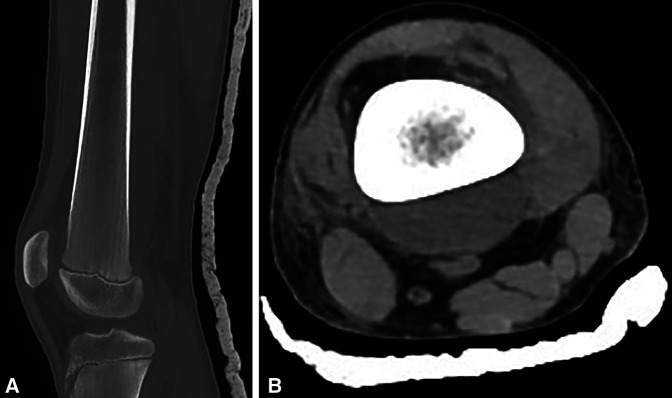
CT scan showing sagittal (**A**) and transverse (**B**) views of the distal femur. A medium-density structure in the posterior surface is identified.

A CT scan of the right knee illustrated a medium-density structure at the posterior surface of the distal femur, possibly full of fluid, without any bone erosion (Figure 3, A and B). In the scanogram, the Erlenmeyer flask deformity of the distal femur was also recognized bilaterally.

An abdominal ultrasonography was also conducted. The only noteworthy finding was spleen size near the highest normal values (nv), measuring 12.2 cm.

A Tc^99m^-HDP bone scan was conducted, where a “hot spot” at the posterior surface of the right distal femur with a “cold” center was detected. Osteomyelitis with sequestrum was the most likely diagnosis; however, a rapidly progressive tumor could not be excluded.

Because of all these findings, an open biopsy of the distal femoral lesion was conducted. The specimen was sent for cultures and histologic examination. The cultures were negative for aerobic and anaerobic bacterial growth. The patient was discharged from the hospital and prescribed antibiotics (cefixime and clindamycin). A week later, he was examined at the outpatient clinic and his symptoms had markedly improved, with slightly painful weight bearing, no edema, and full range of knee motion.

**Figure 4:**
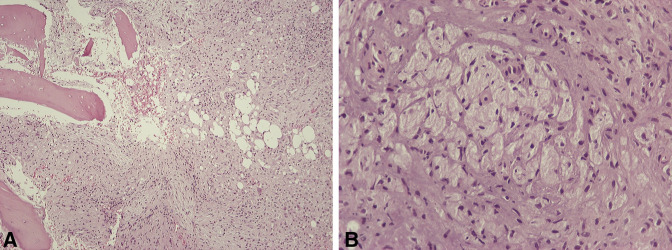
Images showing histologic findings of the bone biopsy specimen: (**A**) On low-power examination, a mixed inflammatory cell infiltrate, accompanied by fibroblastic reaction, is found in the bone marrow. (B) On high-power examination, a cluster of histiocytes with “crinkled-paper” cytoplasm is seen, suggesting the diagnosis of Gaucher disease. **A** and **B**, Hematoxylin-eosin stain (**A**: ×100, **B**: ×400)

Histologic examination of the bone biopsy specimen revealed mixed inflammatory infiltrates in the marrow spaces, composed of histiocytes, lymphocytes, and neutrophils (Figure 4, A). These were accompanied by a fibroblastic reaction, necrobiotic material, loss of osteocytes, and bone resorption. A cluster of histiocytes with features suggestive of Gaucher cells was noticed (Figure 4, B). Microorganisms were not identified on PAS, Giemsa, and Ziehl-Neelsen stains. Therefore, a histologic diagnosis of an inflammatory process was made, and the possibility of underlying GD was considered.

Additional investigation was decided, although the patient's clinical status had fully improved, because it was important to confirm or reject the possible diagnosis of GD. First, the serum enzyme chitotriosidase was measured because it is increased in patients with GD. A sample was sent to a laboratory specialized in GD diagnosis, and a very high value of 6696 nmol/mL/h (nv: 0 to 150 nmoles/mL/h) was found.

Subsequently, the enzyme β-glucocerebrosidase was measured, and the result of 4 nmoles/mg of protein/h (nv: 6 to 23 nmoles/mg of protein/h), that is, a decreased value, confirmed the diagnosis of GD. DNA testing for the mutation of the GBA gene was also done. PCR revealed two heterozygous mutations, N370S and L444P. Thus, the diagnosis of GD was confirmed, and the patient was referred to a pediatric hematologist for follow-up and treatment.

**Figure 5:**
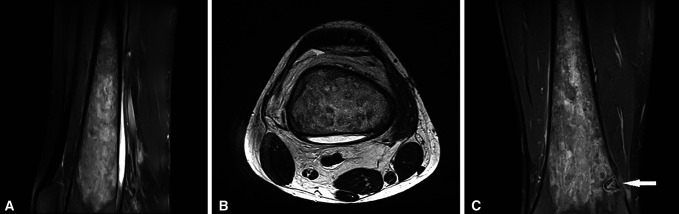
MRI of the right knee 2 months after the onset of symptoms. Distal femur sagittal (**A**) and transverse (**B**) views, which show decreased fluid collection on the posterior surface of the distal femur and decreased soft-tissue edema. Distal femur coronal (**C**) view shows intense bone marrow heterogeneity and the tunnel of the previous open biopsy procedure (white arrow).

Finally, 2 months after the onset of symptoms, the patient underwent a new MRI which confirmed a decrease in the quantity of fluid at the posterior aspect of the distal femur, decreased soft-tissue edema, and residual elevated periosteum. However, more distinct heterogeneity of the bone marrow was obvious (Figure 5, A–C).

## Discussion

We presented a rare case of GD in an adolescent manifesting with knee pain as the first symptom. The persistence of symptoms necessitated additional investigation, first with MRI and subsequently with biopsy, which introduced the suspicion of the disease. The diagnosis of GD was confirmed by the disease-specific blood test results and DNA analysis.

GD is a rare autosomal recessive genetic disorder. It is caused by the functional deficiency of β-glucocerebrosidase, an enzyme that hydrolyzes glucocerebroside (also called glucosylceramide) into ceramide and glucose. Glucocerebroside's accumulation in macrophages causes characteristic changes in their appearance. The modified macrophages are called “Gaucher cells.” However, similar cells, the “pseudo-Gaucher cells,” may appear in other conditions, such as sickle cell anemia, acute lymphoblastic leukemia, multiple myeloma, myelodysplasia, Hodgkin lymphoma, thalassemia, and disseminated mycobacterial infection. Gaucher and pseudo-Gaucher cells can be differentiated by iron staining, where the former are well stained in contrast to the latter, and with examination under the electron microscope, which demonstrates the characteristic tubular cytoplasmic inclusions of Gaucher cells.^6,7^ In our case, the pathologist identified a cluster of histiocytes with features of Gaucher cells in the bone biopsy specimen, and this was crucial for additional laboratory investigation and final confirmation. Nonetheless, biopsy is not usually necessary for the diagnosis of the disease.

The clinical presentation of GD includes a wide variety of symptoms and signs. Hepatomegaly and splenomegaly are common manifestations of GD. The latter is associated with hypersplenism and consequential thrombocytopenia, which may result in bleeding disorders.^8^ The spleen size in our case was near the upper limits; still, no thrombocytopenia was found.

Bone involvement of GD includes alterations of both the mineralized components and the bone marrow. As Gaucher cells gradually replace the normal mesenchymal cells of the bone marrow, osteoblastic and endothelial cell function and hematopoiesis are compromised. This may result in abnormalities in bone formation and remodeling, manifested as the long bone Erlenmeyer flask deformity, osteoporosis, bone infarction, osteonecrosis, and anemia. Bone crisis, as a result of ischemia, presents with acute, severe pain, usually around the joints.^9^ In our case, the complete blood count revealed anemia and knee radiograph demonstrated the Erlenmeyer flask deformity of the distal femur. These findings suggested the need of additional investigation. In addition, after making the diagnosis, we concluded that the persistent periarticular knee pain, which was the main symptom of our patient, was the result of a bone crisis.

Neurologic symptoms in GD include a variety of manifestations. Central nervous system symptoms are usually present in patients with type 3 GD, manifested as seizures or epilepsy. Peripheral neuropathy and parkinsonism are associated with tremor, decreased finger dexterity, instability, and deafness and may be sometimes recognized in type 1 disease.^10^ Patients with peripheral neuropathy may also feel pain, mainly on the extremities, because of the setup of a modified sensation. It is important to differentiate the pain origin because bone pain suggests the need of enzyme replacement therapy (ERT) modification while neurologic pain responds well to analgesics.^11^ In some cases, ocular involvement with horizontal supranuclear gaze palsy has been reported.^12^ In addition, accumulation of Gaucher cells in the bone marrow and hypersplenism may cause inhibition of cytogenesis and progressive cytopenia resulting in myelodysplastic syndrome. This is the reason why a bone marrow examination is important in such patients.^13^ Rarely, the lungs, kidneys, skin, myocardium, and pancreas may be involved.^8^ In our case, no symptom other than knee pain was reported. However, the presence of anemia indicated the need for future bone marrow function monitoring to early diagnose the possible development of myelodysplastic syndrome.

The diagnosis of GD is mainly based on the laboratory findings. Yet, clinical signs, such as hepatosplenomegaly and distinct bone pathology; neurological symptoms; and blood test abnormalities, such as thrombocytopenia or anemia, may guide to additional investigation and correct diagnosis.^5^ Bone marrow biopsy is not necessary for the diagnosis of GD; still, it is useful for differential diagnosis of patient's early symptoms. In addition, the discovery of Gaucher cells is important for the course of the investigation. Furthermore, many serum proteins may be elevated. Chitotriosidase, angiotensin-converting enzyme, tartrate-resistant acid phosphatase, and CCL18 are included. Yet, GD is confirmed by the measurement of decreased β-glucocerebrosidase activity in peripheral blood leukocytes and DNA analysis, which may detect relevant mutations of the GBA gene; these are useful for family screening too.^14^ The most common variants are L44P, associated with all three types of GD, and N307S, encountered in type 1, commonly in the Ashkenazi Jewish population.^15^ Heterozygous mutations L444P and N370S were found in our patient, resulting in a phenotype of type 1 disease.

Treatment options of GD include two major categories: ERT and substrate reduction therapy (SRT). ERT corrects the underlying deficiency and is effective in all age groups, administrated before irreversible visceral and bone complications. However, because the available agents do not pass the blood-brain barrier, they have no effect in controlling the central nervous system symptoms of GD types 2 and 3. Contrarily, oral SRT acts by inhibiting glucocerebroside synthesis and is usually used in patients unwilling to receive intravenous therapy or with a history of adverse events after ERT. SRT can be administrated in combination with the latter for the improvement of neuropathic features. Apart from these two options, bone marrow transplantation, especially before ERT; splenectomy after symptomatic splenomegaly; and orthopaedic surgeries, mainly arthroplasty after arthritis because of osteonecrosis, may be necessary as additional procedures. Finally, as in every hereditary disease, gene therapy and genome editing is the future for the treatment of this autosomal recessive genetic disease.^16^ After pediatric hematologist assessment, our patient has been administered ERT.

Bone pain may be an early manifestation in patients with GD, especially in childhood and adolescence. Because it generally occurs along with elevated inflammatory markers and signs of local inflammation, a possible misdiagnosis of osteomyelitis may delay adequate therapy and allow progression of bone involvement.^17^ The differential diagnosis between these two conditions is challenging. During bone crisis in GD, MRI illustrates high signal intensity of the bone marrow in T2-weighted images because of edema; however, osteomyelitis has similar imaging.^18^ Most frequently, the only clue in favor of GD is negative cultures after a biopsy. Still, diagnostic suspicion of GD may prompt measurement of β-glucocerebrosidase, which in combination with DNA analysis can confirm the diagnosis, without any orthopaedic intervention.^19^

## Conclusion

GD is a hereditary lysosomal storage disorder resulting in β-glucocerebrosidase deficiency. It usually presents with visceral and skeletal abnormalities. Bone manifestations are frequently expressed as acute disabling pain, usually near the joint. This is the main symptom of patients with GD who visit orthopaedic surgeons for investigation and adequate treatment. In the event of nontraumatic pain with acute onset, except for osteomyelitis and malignancy, it is important to also look for more unusual diseases, when the clinical and laboratory findings are controversial. GD is such a rare disease, and it is important to be successfully diagnosed because early therapeutic agent introduction is crucial for the final outcome and life expectancy.

## References

[1] Riboldi GM Di Fonzo AB: GBA, Gaucher disease, and Parkinson's disease: From genetic to clinic to new therapeutic approaches. Cells 2019;8:E364. 31010158 10.3390/cells8040364 PMC6523296

[2] Nalysnyk L Rotella P Simeone JC Hamed A Weinreb N: Gaucher disease epidemiology and natural history: A comprehensive review of the literature. Hematology 2017;22:65-73. 27762169 10.1080/10245332.2016.1240391

[3] Rosenbloom BE Weinreb NJ: Gaucher disease: A comprehensive review. Crit Rev Oncog 2013;18:163-175. 23510062 10.1615/critrevoncog.2013006060

[4] Dandana A Ben Khelifa S Chahed H Miled A Ferchichi S: Gaucher disease: Clinical, biological and therapeutic aspects. Pathobiology 2016;83:13-23. 26588331 10.1159/000440865

[5] Baris HN Cohen IJ Mistry PK: Gaucher disease: The metabolic defect, pathophysiology, phenotypes and natural history. Pediatr Endocrinol Rev 2014;12(Suppl 1):72-81. 25345088 PMC4520262

[6] Bain BJ Lee L: Pseudo-Gaucher cells in sickle cell anemia. Am J Hematol 2010;85:435. 20143404 10.1002/ajh.21647

[7] Goren Sahin D Uskudar Teke H Karagulle M : Gaucher cells or pseudo-Gaucher cells: That's the question. Turk J Haematol 2014;31:428-429. 25541666 10.4274/tjh.2014.0019 PMC4454064

[8] Stirnemann J Belmatoug N Camou F : A review of Gaucher disease pathophysiology, clinical presentation and treatments. Int J Mol Sci 2017;18:E441. 28218669 10.3390/ijms18020441 PMC5343975

[9] Hughes D Mikosch P Belmatoug N : Gaucher disease in bone: From pathophysiology to practice. J Bone Miner Res 2019;34:996-1013. 31233632 10.1002/jbmr.3734 PMC6852006

[10] Pastores GM: Neuropathic Gaucher disease. Wien Med Wochenschr 2010;160:605-608. 21221912 10.1007/s10354-010-0850-x

[11] Devigili G De Filippo M Ciana G : Chronic pain in Gaucher disease: Skeletal or neuropathic origin? Orphanet J Rare Dis 2017;12:148. 28859662 10.1186/s13023-017-0700-7 PMC5580212

[12] Eghbali A Hassan S Seehra G FitzGibbon E Sidransky E: Ophthalmological findings in Gaucher disease. Mol Genet Metab 2019;127:23-27. 31047801 10.1016/j.ymgme.2019.02.002

[13] Ruchlemer R Mittelman M Zimran A: Gaucher disease, myelodysplastic syndrome and ICUS. Blood Cell Mol Dis 2020;80:102373. 10.1016/j.bcmd.2019.102373 31718920

[14] Mistry PK Cappellini MD Lukina E : A reappraisal of Gaucher disease-diagnosis and disease management algorithms. Am J Hematol 2011;86:110-115. 21080341 10.1002/ajh.21888 PMC3058841

[15] Gary SE Ryan E Steward AM Sidransky E: Recent advances in the diagnosis and management of Gaucher disease. Expert Rev Endocrinol Metab 2018;13:107-118. 30058864 10.1080/17446651.2018.1445524 PMC6129380

[16] Revel-Vilk S Szer J Mehta A Zimran A: How we manage Gaucher disease in the era of choices. Br J Haematol 2018;182:467-480. 29808905 10.1111/bjh.15402

[17] Marcucci G Zimran A Bembi B : Gaucher disease and bone manifestations. Calcified Tissue Int 2014;95:477-494. 10.1007/s00223-014-9923-y 25377906

[18] Simpson WL Hermann G Balwani M: Imaging of Gaucher disease. World J Radiol 2014;6:657-668. 25276309 10.4329/wjr.v6.i9.657 PMC4176783

[19] Oliveri B González DC Ferrari E: Bone symptoms can be an early manifestation of Gaucher disease implications for diagnosis. Endocr Metab Sci 2020;1:100050.

